# Thickness of retina and choroid in the elderly population and its association with Complement Factor H polymorphism: KLoSHA Eye study

**DOI:** 10.1371/journal.pone.0209276

**Published:** 2018-12-31

**Authors:** Na-Kyung Ryoo, Seong Joon Ahn, Kyu Hyung Park, Jeeyun Ahn, Jiyeong Seo, Ji Won Han, Ki Woong Kim, Se Joon Woo

**Affiliations:** 1 Department of Ophthalmology, Seoul National University College of Medicine, Seoul National University Bundang Hospital, Seongnam, Korea; 2 Department of Ophthalmology, Veterans Health Service Medical Center, Seoul, Korea; 3 Department of Ophthalmology, Hanyang University, Seoul, Korea; 4 Department of Ophthalmology, Seoul Metropolitan Government Seoul National University Boramae Medical Center, Seoul, Korea; 5 Department of Psychiatry, Gyeongsang National University Hospital Changwon Hospital, Changwon, Korea; 6 Department of Neuropsychiatry, Seoul National University Bundang Hospital, Seongnam, Korea; 7 Department of Neuropsychiatry, Seoul National University College of Medicine, Seoul, Korea; 8 Department of Brain and Cognitive Science, Seoul National University College of Natural Sciences, Seoul, Korea; University of Manchester, UNITED KINGDOM

## Abstract

**Purpose:**

To analyze the associations of retinal and choroidal thickness on enhanced-depth imaging optical coherence tomography (EDI-OCT) with clinical, ophthalmic and genetic factors in the normal elderly population (aged 65 years or older).

**Methods:**

In this prospective, population-based cohort study, people aged 65 years or older were enrolled in the baseline study of the Korean Longitudinal Study on Health and Aging (KLoSHA) Eye Study. All participants underwent spectral domain-OCT scan using the EDI technique. A topographic map of the retina was obtained and subfoveal choroidal thickness (SFCT) was measured manually. Blood samples from all subjects were genotyped for major age-related macular degeneration (AMD)-associated single nucleotide polymorphisms (SNPs) the major AMD-associated SNPs; *CFH* Y402H rs1061170, *CFH* I62V rs800292, *ARMS2* A69S rs10490924. A statistical analysis was conducted to compare the retinal thickness, choroidal thickness, and AMD risk genotypes.

**Results:**

Among the three hundred eighty people enrolled, the mean age was 76.6 years (range 65–99 years). Factors that showed correlation with either tomographic retinal parameters, retinal nerve fiber layer, or SFCT, were age and gender. Significant age-related decrease in thickness was observed in the RNFL, mean central thickness (MCT) and SFCT. Gender differences existed in central foveolar thickness (CFT) and MCT, where it was thicker in men. While chorioretinal parameters were not related with other genotypes, *CFH* rs1061170 risk genotype was significantly associated with thin SFCT. The group containing the AMD- risk allele (CT) had a 14.7% reduction in the SFCT compared to the non-risk TT group.

**Conclusions:**

In addition to the well-known association with AMD, *CFH* rs1061170 is a significant genetic risk factor associated with choroidal thinning in normal eyes of the elderly population. Such findings may provide further insight into the pathogenesis of age-related macular degeneration as well as normal aging. In addition, our study provides the first normative data on retinal and choroidal thickness in population-based aged groups with a mean age over seventy-five.

## Introduction

Age-related macular degeneration (AMD) is a leading cause of visual impairment in the elderly population, especially those over 60 years of age [1]. Under the current circumstances of population exponential ageing, the global burden of AMD has been projected to reach approximately 196 million in 2020 and 288 million in 2040 [2]. Despite the rapidly rising prevalence of AMD with the increase in the aging population, the pathophysiology underlying AMD remains unclear.

AMD is thought to be a multifactorial disease, with genetic factors playing a role in the development of the disease [3–5]. Complement Factor H (*CFH*) is one of the major genes that accounts for the genetic basis of AMD. *CFH* along with age-related maculopathy susceptibility2 (*ARMS2*) are considered to be responsible for nearly 80% of the genetic risk of AMD. In our previous study on Korean AMD patients and controls, significant associations of *ARMS2* rs10490924 SNP and *CFH* rs800292 with AMD were found, while *CFH* Y402H variant (rs1061170), which is the major genetic variant among Caucasians, showed insignificant association [6]. Such genetic alterations appear important in determining AMD risk; however, the underlying pathology from a genetic predisposition aspect is less evident. Possible associations between choroidal thickness and AMD have been explored in multiple studies [7, 8]. Chung et al. reported significant choroidal thinning in eyes with neovascular AMD and early AMD compared with normal subjects [7].

Polypoidal choroidal vasculopathy (PCV) being one of the most common subtypes of neovascular AMD in Asia, previous genetic studies also focused on the relationship of choroidal thickness in PCV. Some reported that subfoveal choroidal thickness differs according to I62V polymorphism in the *CFH* gene in typical AMD and PCV patients [9]. Yoneyama et. al. reported that subfoveal choroidal thickness in eyes with treatment-naive PCV were associated with both the G allele of *ARMS2* rs10490924 and the T allele of *CFH* rs1329428. [10]

Whether choroidal thinning is a consequence or an underlying trigger of the disease is unclear. Several reports on retinal and choroidal thickness in the normal population exist [11–13]. However, limitations such as insufficient numbers of subjects, non-population-based studies, and lack of enhanced-depth imaging hinder the impact of the studies. Direct associations between choroid or retinal structures and genetic background, especially in the healthy aging population remain unknown.

In this study, we aimed to evaluate choroid and retinal thickness—defined by OCT parameters—and their association with clinical variables as well as major AMD-related genes in normal eyes of the elderly population, based on a population-based cohort study.

## Methods

This study was conducted as a part of the KLoSHA study (Korean Longitudinal Study on Health and Aging), which was designed as a population-based prospective cohort study on health, aging, and common geriatric diseases in the Korean elderly aged 65 years and over [14].

KLoSHA was conducted in the city of Seongnam, one of the largest satellite cities of Seoul, South Korea. Located in the outskirts of Seoul, Seongnam had a population of 996,524 in 2010, and 84,043 (8.4%) of the population was older than 65 years of age [15].

The subjects of KLoSHA also participated in the AMD gene study in Seoul National University Bundang Hospital and thus their gene and ophthalmic data could be used altogether. The KLoSHA Eye Study was initiated with the objective to estimate the prevalence, incidence and progression of common geriatric eye diseases, and to obtain normative values of the ocular structure in the elderly population as well as to determine the risk factors associated with geriatric-associated pathological changes. The KLoSHA Eye Study was conducted from September 2010 through September 2011. A total of 380 population-based randomly sampled people aged 65 years or more were enrolled.

The Institutional Review Board of Seoul National University Bundang Hospital approved this study, and the study adhered to the tenets of the Declaration of Helsinki. Written informed consent was obtained from all subjects before participation in the study.

**Fig 1:**
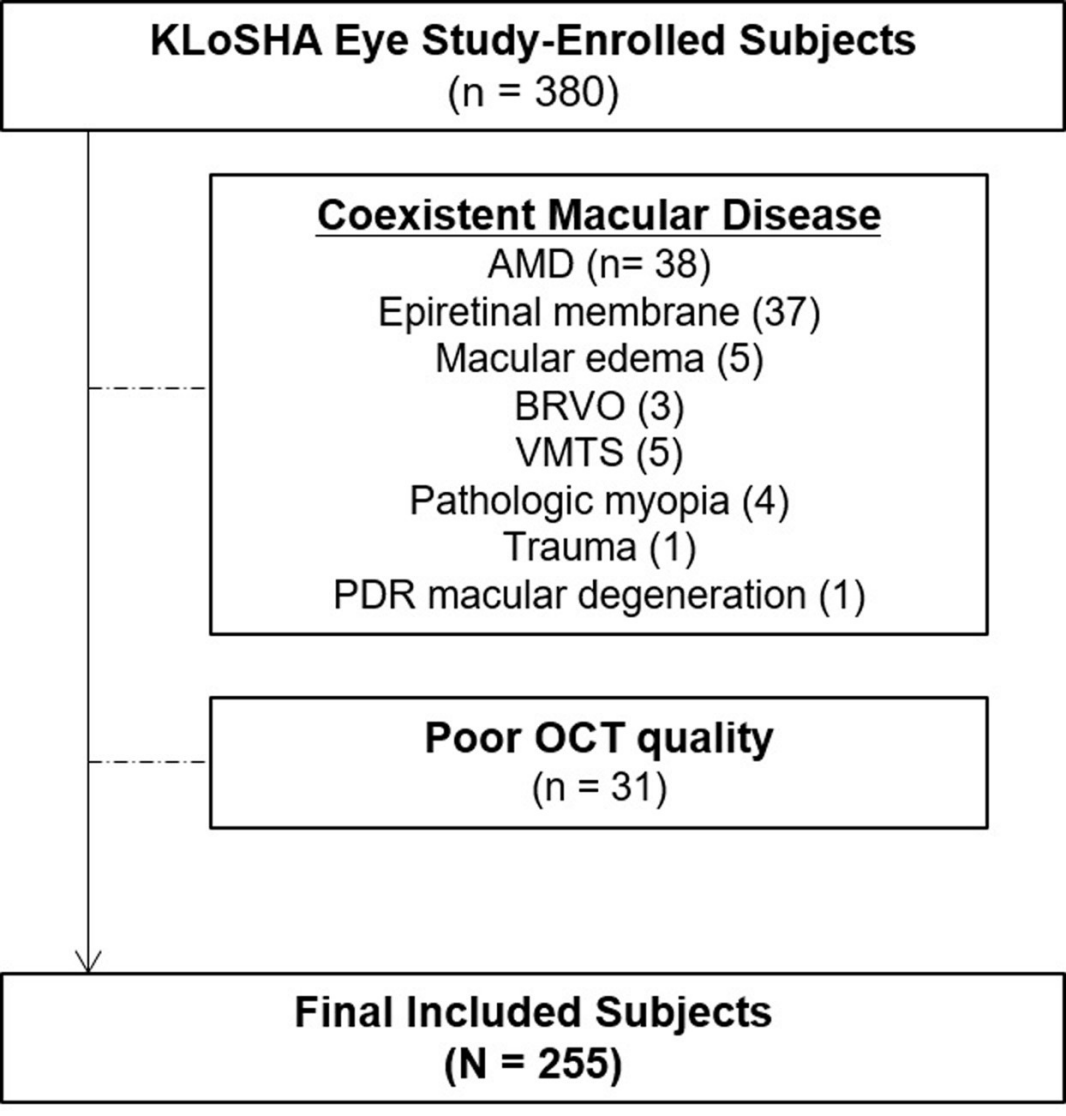
Flow diagram of the subject selection process. KLoSHA = Korean Longitudinal Study on Health and Aging; AMD = age-related macular degeneration, BRVO = branch retinal vein occlusion; VMTS = vitreomacular traction syndrome; PDR = Proliferative Diabetic Retinopathy; OCT = Optical Coherence Tomography.

All subjects underwent general health condition evaluations with laboratory tests, and ophthalmologic exams. Ophthalmologic exams included best-corrected visual acuity (BCVA), intraocular pressure, slit lamp examination, auto-refractometry, axial length measurement using optical biometry (IOLMaster; Carl-Zeiss Meditec, Dublin, CA), color fundus photography and spectral-domain optical coherence tomography (SD-OCT) using an enhanced-depth imaging (EDI) technique. Exclusion criteria included poor OCT quality, coexistent macular diseases, e.g. AMD, epiretinal membrane, macular edema, branch retinal vein occlusion (BRVO), vitreomacular traction syndrome (VMTS), pathologic myopia, traumatic maculopathy, macular degeneration due to proliferative diabetic retinopathy, and glaucoma. The final analysis was performed in 255 participants out of 380 initially enrolled subjects (Fig 1). Randomization was done to determine the laterality of the eye for analysis.

**Fig 2:**
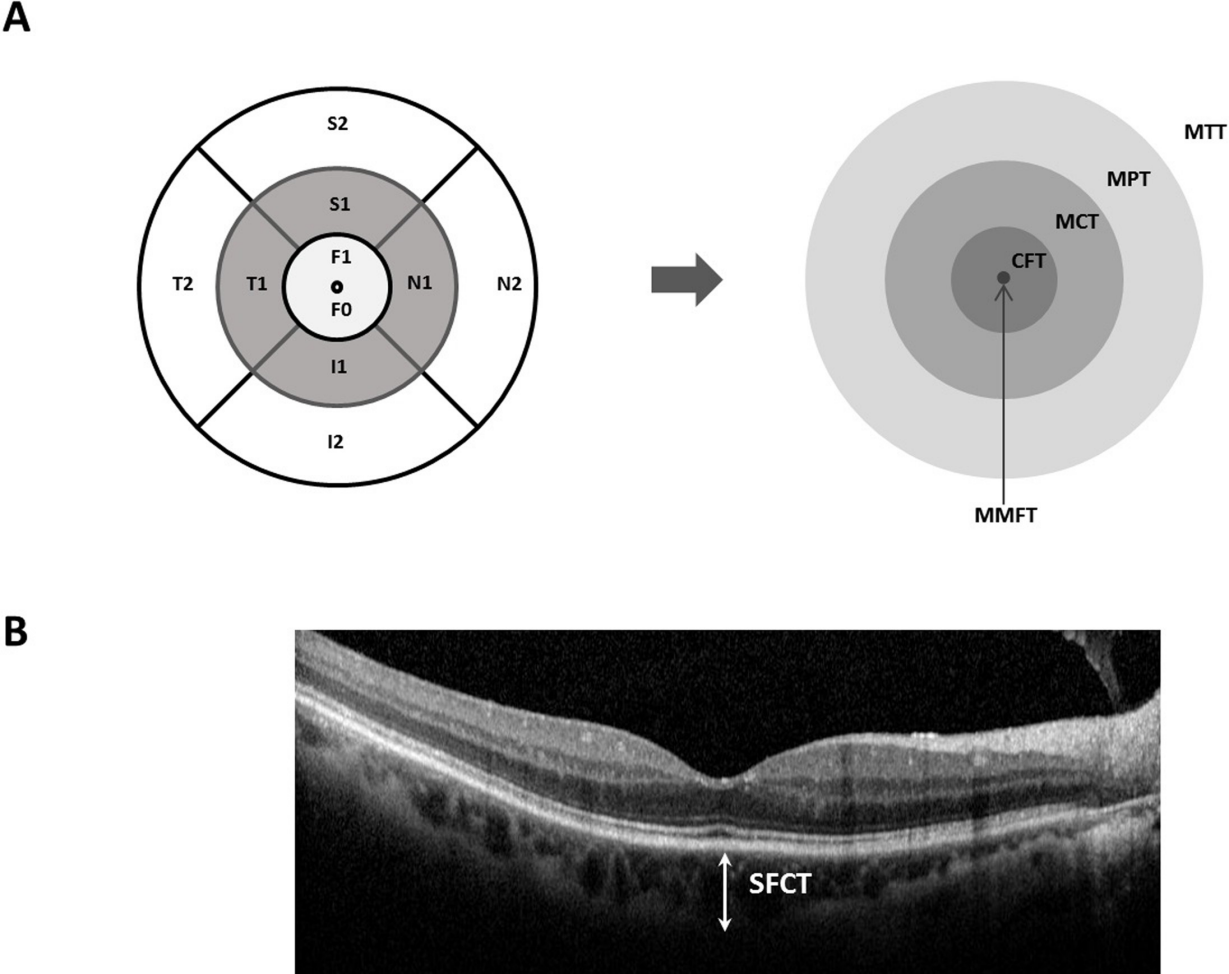
Diagram of the retinal and choroidal thickness measurement areas obtained with SD-OCT (EDI) System. **A, Diagram of the macular areas on the SD-OCT scan.** The 9 standard ETDRS regions are converted into areas of interest, i.e. MMFT, CFT, MCT, MPT, MTT. Each ring, centered on the fovea, has a diameter of 6, 3, 1 millimeters, respectively. MMFT = mean minimal foveolar thickness = F0; CFT = central foveolar thickness = F1, MCT = mean central thickness = (S1+N1+I1+T1) / 4; MPT = mean peripheral thickness = (S2+N2+I2+T2) / 4; MTT = mean total region thickness = (F1+ S1~T1 + S2~T2) / 9; S = superior; I = inferior; N = nasal; T = temporal; ETDRS = Early Treatment Diabetic Retinopathy Study. **B. Representative EDI image used in measuring SFCT.** SFCT = subfoveal choroidal thickness.

Retinal and choroidal images were obtained in both eyes with the Spectral-domain OCT (Spectralis, Heidelberg Engineering, Germany). The macula protocol, consisting of a raster-scan composed of 31 horizontal lines centered on the fovea, was performed with 25 frames averaged for each OCT B-scan to obtain improved image quality. Horizontal and vertical images crossing the fovea were also obtained using the enhanced-depth imaging (EDI) technique. Thickness and volume maps were obtained based on the 9 macular fields introduced by the Early Treatment Diabetic Retinopathy Study (ETDRS) group [16, 17]. The 9 fields are represented by 3 concentric rings centered on the fovea measuring 1 mm (inner ring), 3 mm (middle ring) and 6 mm (outer ring) in diameter. The innermost point represents the foveola and the inner ring is the fovea, while the 3-mm and 6-mm rings are further divided into four equal regions. Each region was summated and divided to represent the mean central thickness, mean peripheral thickness, and mean total thickness (Fig 2). Mean central thickness (MCT) and mean peripheral thickness (MPT) were defined by averaging middle (MCT = (S1+N1+I1+T1)/4) and outer (MPT = (S2+N2+I2+T2)/4) four quadrants. Mean total region thickness (MMT) was calculated by averaging all 9 macular regions within the 6-mm ring (MMT = F1+S1+N1+I1+T1+S2+N2+I2+T2)/9) [18, 19]. Average retinal nerve fiber layer thickness was obtained from a built-in automated program. Subfoveal choroidal thickness (SFCT) was determined as the averaged value measured manually using EDI images through the center of the fovea by two independent retina specialists (N.K.R. and S.J.A.).

Blood samples from all subjects were genotyped for the major AMD-associated SNPs: *CFH* Y402H rs1061170, *CFH* I62V rs800292, *ARMS2* A69S rs10490924. The selection of SNPs was based on the prior report on Korean AMD patients [6].

Statistical analyses were performed using SPSS software version 23.0 (IBM Inc, Chicago, Il.). Independent sample T-test, chi-square test and Fisher’s exact test were used to compare parameters. Linear regression analysis was used to determine an association of RNFL, MCT and SFCT with age. Multi-variant analysis with age, gender and axial length as independent variables was also performed to determine the variations in thickness measurements when controlled for the factors mentioned above. ANOVA test was used for genetic analysis. P values less than 0.05 were considered to indicate statistically significant differences. Instead of applying a strict Bonferroni correction we aimed for an exploratory analysis applying such p-value [20].

## Results

The mean age of the 255 elderly subjects was 76.6 years (range 65–99 years). One hundred twenty-six subjects (49%) were males while 129 subjects (51%) were females. The mean ages of males and females were 76.3 years and 76.9 years respectively (*p* = 0.4). In addition, the mean axial lengths were 23.5±0.9 in males and 22.8±0.8 in females (*p*<0.05). The refractive error ranged from -2.88 diopters (D) to +5.63 D with no gender-specific significance (*p* = 0.564). Detailed demographics of the subjects are given in Table 1.

**Table 1:** Demographic data and OCT measurements of the subjects.

**Demographics**		
Total (N)		255
Age (Avg.(SD))		76.6 (6.2)
Sex (M:F)		126:129
HTN (N,%)	yes	32 (13)
	no	223 (88)
Phakia (N,%)	yes	219 (86)
	no	35 (14)
BCVA_logMAR (range)		-0.85~ +0.70
RefErr (Diopter range)		-2.88~+5.63
AXL (Avg.(SD))		23.2 (0.9)
(range)		20.2 ~ 25.4
**OCT parameters (Avg. (SD))**		**μm**
MMFT		217 (21)
CFT		265 (24)
MCT		327 (20)
MPT		289 (59)
MTT		303 (30)
RNFL		95 (12)
SFCT		182 (69)

HTN = Hypertension; BCVA = Best-corrected Visual Acuity; RefErr = Refractive Error; D = Diopters; AXL = Axial Length; MMFT = mean minimal foveolar thickness; CFT = central foveolar thickness; MCT = mean central thickness; MPT = mean peripheral thickness; MTT = mean total region thickness; RNFL = retinal nerve fiber layer; SFCT = subfoveal choroidal thickness.

Using the ETDRS map, minimal foveolar thickness for all subjects was 217±21 (mean±SD) μm, central foveolar thickness 265±24 μm, mean central thickness 327±20 μm, mean peripheral thickness 289±59 μm, mean total region thickness 303±30 μm, retinal nerve fiber layer thickness 95±12 μm, and subfoveal choroidal thickness 182±69 μm (Table 1).

**Fig 3:**
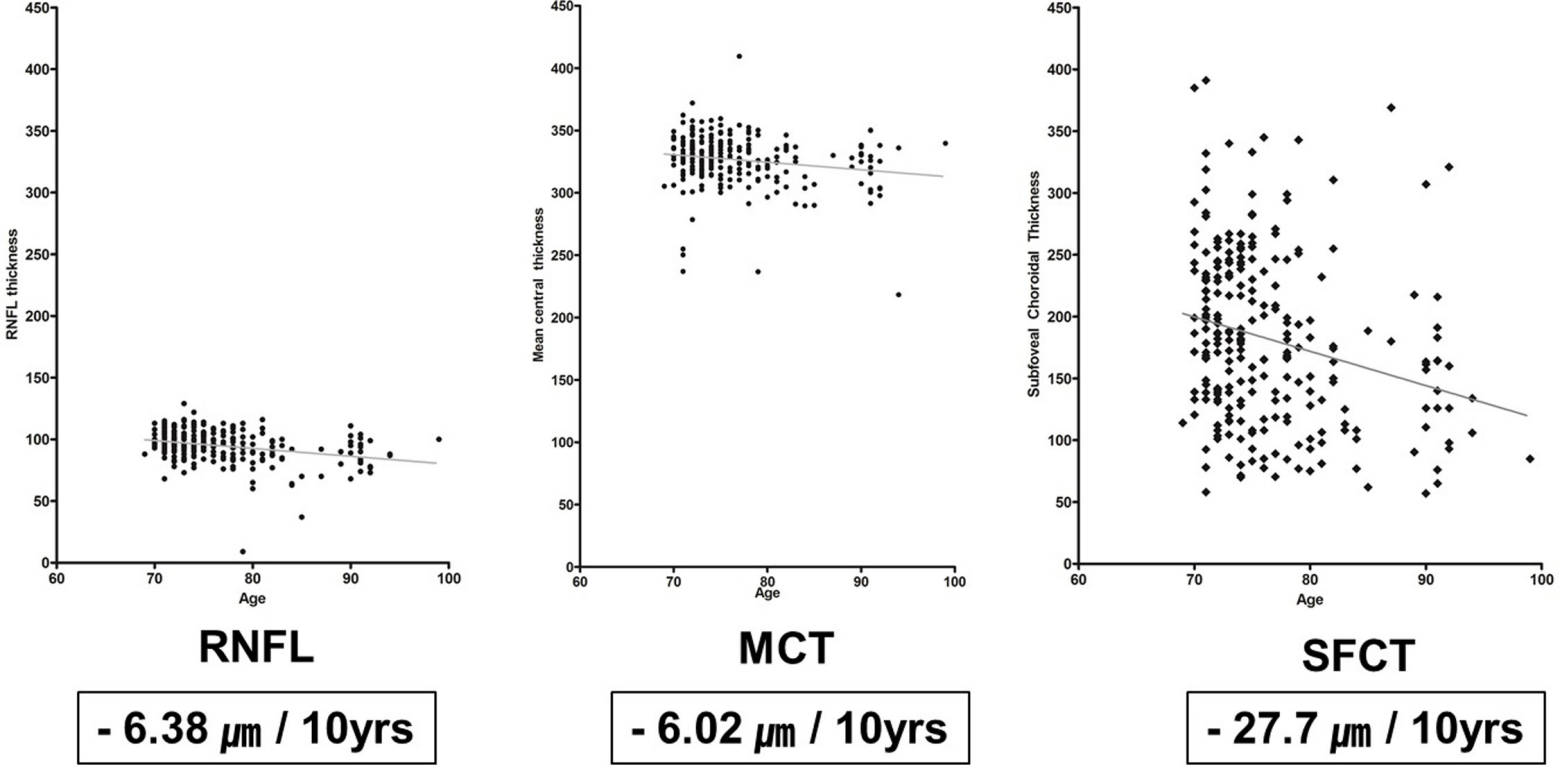
Scatter plot and the regression line of RNFL, MCT, SFCT against age. RNFL = retinal nerve fiber layer; MCT = mean central thickness; SFCT = subfoveal choroidal thickness.

Correlations among OCT parameters and age, gender, hypertension, phakia and axial length are presented in Table 2. Factors that showed correlation with either tomographic retinal parameters, retinal nerve fiber layer, or SFCT, were age and gender. Significant age-related decrease in thickness was observed in the RNFL, MCT and SFCT via regression analysis (*p* = <0.0001, 0.003, and <0.0001, respectively; r^2^ = 0.09, 0.04 and 0.07 respectively) (Fig 3). Examining different age groups, the RNFL thickness decreased at a rate of 6.38 μm over a time period of 10 years, while the MCT decreased 6.02 μm over 10 years, and SFCT decreased most rapidly at a rate of 27.7 μm per 10 years (Fig 3).

**Table 2:** Correlation between OCT parameters and age, gender, hypertension, presence of phakia and axial length.

		Age		Gender*			HTN*	Phakia*	AXL^†^	
OCT parameters (Avg. (SD))		Correlation coefficient	*P*	Male	Female	*P*	*P*	*P*	Correlation coefficient	*P*
MMFT	217 (21)	0.025	0.694	220 (18)	215(24)	0.372	0.726	0.148	-0.043	0.506
CFT	265 (24)	-0.025	0.698	275(10)	261(9)	0.005^§^	0.604	0.529	0.044	0.496
MCT	327 (20)	-0.157	0.012^‡^	335(21)	320 (11)	0.021^‡^	0.216	0.806	0.045	0.483
MPT	289 (59)	-0.056	0.378	290(62)	287(57)	0.275	0.343	0.761	-0.112	0.081
MTT	303 (30)	-0.107	0.088	305(31)	301(28)	0.527	0.569	0.672	-0.072	0.263
RNFL	95 (12)	-0.300	<0.01^§^	94(11)	96(13)	0.438	0.181	0.276	-0.090	0.166
SFCT	182 (69)	-0.248	<0.01^§^	184(69)	179(69)	0.309	0.509	0.101	-0.071	0.272

HTN = Hypertension; AXL = Axial length; MMFT = mean minimal foveolar thickness; CFT = central foveolar thickness; MCT = mean central thickness; MPT = mean peripheral thickness; MTT = mean total region thickness; RNFL = retinal nerve fiber layer; SFCT = subfoveal choroidal thickness.

^*^Chi square / Fisher-exact Test;

^†^ Pearson’s chi-square test;

^‡^ P-value < 0.05,

^§^P-value <0.01

**Fig 4:**
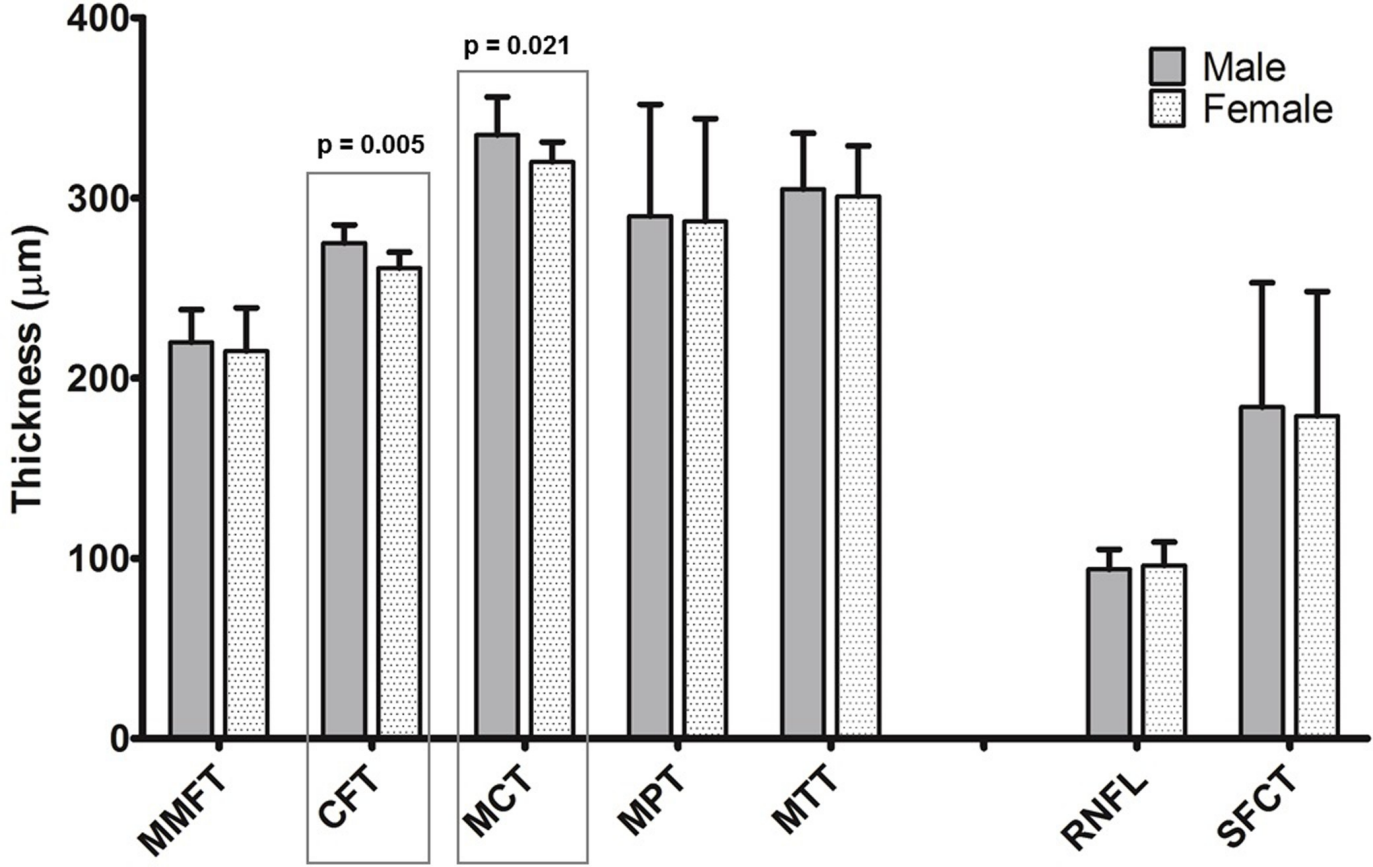
Retinal and choroidal thickness measurements by gender. **Error bars represent standard error of the mean**. MMFT = mean minimal foveolar thickness; CFT = central foveolar thickness; MCT = mean central thickness; MPT = mean peripheral thickness; MTT = mean total region thickness; RNFL = retinal nerve fiber layer; SFCT = subfoveal choroidal thickness;

Central foveolar thickness and mean central thickness differed between genders (CFT: 275 ± 10 μm vs. 261± 9 μm (men vs. women), MCT: 335 ± 21 μm vs. 320 ± 11 μm, p = 0.005 and p = 0.021 respectively), implying that men have a thicker retina within the 3 mm zone than women. The difference was still significant after adjustment of axial length. No gender-specific differences were detected in the MMFT, MPT, MTT, RNFL thickness or subfoveal choroidal thickness (Fig 4). Hypertension, presence of crystalline lens or axial length showed no statistical significance related to retinal or choroidal thickness, after adjustment with age and gender (Table 2).

**Fig 5:**
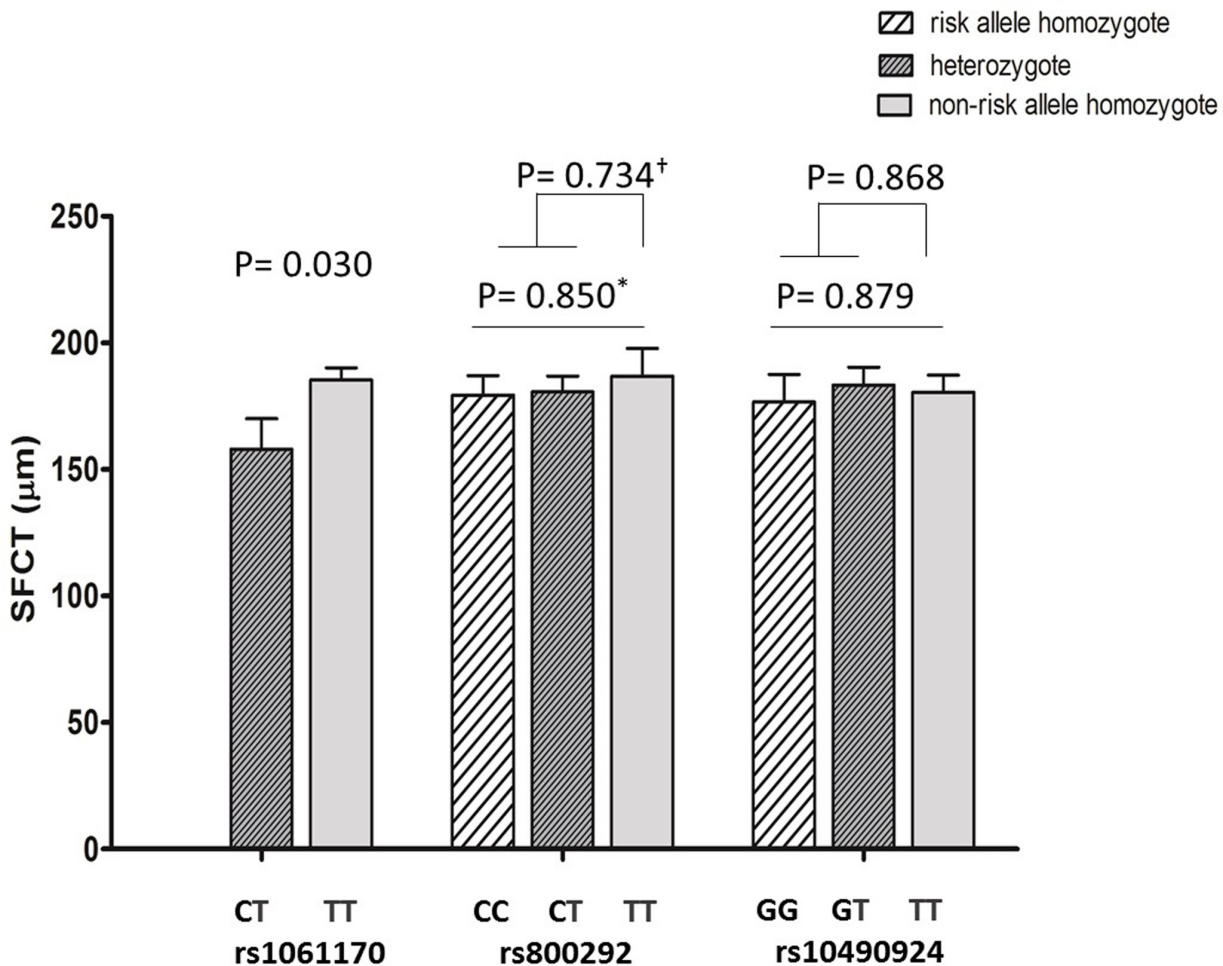
Subfoveal choroidal thickness and its genetic distribution according to polymorphism of complement factor H gene (rs1061170 and rs800292) and age-related maculopathy susceptibility 2 gene (rs10490924). Error bars represent standard error of the mean. ^*^ANOVA analysis; ^†^ ANCOVA analysis. *P*-values after adjustment of age, gender and axial length.

**Fig 6:**
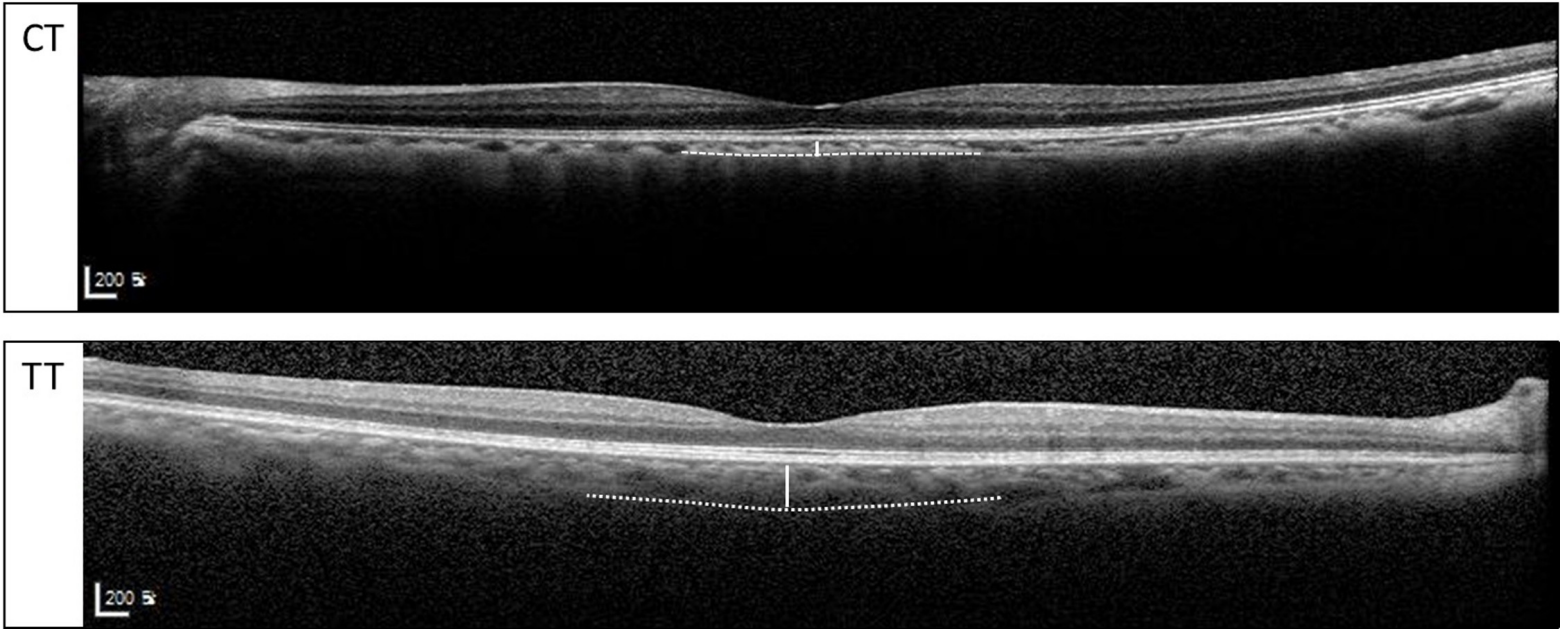
Representative images of subfoveal choroidal thickness measured on SD-OCT (EDI technique) between patients with CT and TT genotypes of CFH gene polymorphism (rs1061170). SD-OCT = spectral-domain optical coherence tomography; EDI = enhanced-depth imaging.

Allelic distributions of each genotype and the thickness values are presented in Table 3 with p-values adjusted for age, gender and axial length. When analyzed according to the presence of a risk allele (C: *CFH* rs800292 and rs1061170, T: *ARMS2* rs10490924), other parameters (MMFT, CFT, MCT, MPT, MTT, and RNFL) are not affected while subfoveal choroidal thickness shows an association with rs1061170 *CFH* risk genotype (Fig 5). Subfoveal choroidal thickness was significantly thinner (158.0±12.0 μm) in subjects with genotype CT than in those with genotype TT (185.3±4.9 μm, p = 0.030). The group containing the risk allele (C) had a 14.7% reduction in the SFCT. Representative EDI-OCT images of high-risk genotype and low-risk genotype patients are shown in Fig 6. The presence of risk alleles in *CFH* rs800292 and *ARMS2* rs10490924 were not associated with the thickness of choroid or the retinal parameters measured.

**Table 3:** Genotype allelic distribution of *CFH* rs1061170, *CFH* rs800292, and *ARMS2* rs10490924 and its relations with retina and choroidal thickness values.

		rs1061170			rs800292				rs10490924			
		CT	TT		CC	CT	TT		GG	GT	TT	
	N	37	194	p-value^*^	72	129	39	p-value^*^	98	107	34	p-value^*^
**MMFT**	Mean	214.5	216.1	0.651	214.2	217.3	219.5	0.358	218.4	217.3	209.9	0.096
	SEM	3.2	1.4		2.5	1.6	3.5		2.0	1.9	3.4	
**CFT**	Mean	265.7	264.1	0.674	265.9	262.6	265.4	0.564	265.8	264.0	259.3	0.359
	SEM	3.9	1.5		2.7	2.0	3.2		2.2	2.3	3.5	
**MCT**	Mean	315.9	325.3	0.354	328.0	319.6	331.4	0.100	319.1	329.2	322.0	0.119
	SEM	9.8	2.1		2.4	3.9	2.1		2.0	1.7	4.8	
**MPT**	Mean	294.5	285.8	0.671	293.8	283.4	286.7	0.567	289.3	286.1	283.6	0.893
	SEM	20.0	3.7		9.4	5.9	2.1		10.3	1.3	2.8	
**MTT**	Mean	300.0	300.8	0.947	305.9	296.8	304.2	0.269	299.4	302.8	298.0	0.771
	SEM	12.2	2.3		4.6	1.1	1.4		6.2	1.3	3.3	
**RNFL**	Mean	93.1	95.8	0.216	94.7	95.1	97.3	0.554	93.3	97.6	93.6	0.036
	SEM	3.1	0.8		1.8	1.1	1.4		1.4	1.1	1.9	
**SFCT**	Mean	158.0	185.3	***0*.*030***	179.3	180.6	186.8	0.850	180.5	183.3	176.7	0.879
	SEM	12.0	4.9		7.7	6.2	10.9		6.7	7.1	10.9	

MMFT = mean minimal foveolar thickness; CFT = central foveolar thickness; MCT = mean central thickness; MPT = mean peripheral thickness; MTT = mean total region thickness; RNFL = retinal nerve fiber layer; SFCT = subfoveal choroidal thickness; CFH = complement factor H, ARMS2 = age-related maculopathy susceptibility 2.

^*^P value from ANOVA test, adjusted for age, gender and axial length.

## Discussion

We investigated the association of retinal and choroidal thickness of normal eyes with clinical, ophthalmic and genetic factors in a population-based cohort of elderly (over 65 years of age) and found significant age-related decreases, differences according to gender and a relationship between choroidal thinning and *CFH* risk genotype.

Our results show a mean minimal foveolar thickness of 217±21 μm, CFT 265±24 μm, MCT 327±20 μm, MPT 289±59 μm, MTT 303±30 μm, RNFL 95±12 μm, and subfoveal choroidal thickness of 182±69 μm in normal eyes of elderly subjects. Direct comparisons with other studies are difficult due to the variability of measurements reported, but mostly due to the lack of studies in such highly aged groups. Our study provides the first normative data and genetic analysis in population-based aged groups with a mean age over seventy-five. Wexler et al. (N = 107, mean age = 42.4 years) reported a CFT of 220±83 μm, MCT 275±12 μm, MPT 272±12 μm and MTT 267±13 μm in a mixed gender group of Norwegians using Stratus OCT [18]. Considering the age-related change and discrepancy between Stratus and Spectralis measurements [21], these values are comparable to our results. Adhi et al (N = 220, mean age = 45.3 years) reported a CFT of 229±20 μm, MCT 290±18 μm, MPT 247±14 μm in Pakistanis using SD-OCT., while Oshitari et al. (N = 30, age 27 ~ 77 years) reported a significantly thicker retinal thickness, within the 3 mm zone in Japanese compared to Caucasians (quadrants sectorial range 262± 12 ~ 274± 12 μm vs. 251±13 ~ 267±16 μm) [22]. Such discrepancies in the thickness of retinal areas reported in previous studies may also be associated with the age or ethnicity of the study populations [16, 18, 22, 23].

There were significant age-related decreases in RNFL, MCT, and SFCT. In our study, the decadal decrease in RNFL thickness and MCT were similar, 6.38 μm and 6.02 μm, respectively, and the subfoveal choroidal thickness decreased 2.77 μm yearly. An inverse correlation of age and RNFL thickness has been suggested by many studies [13, 24–26]. A decadal decrease of 2.24 μm [13] and 3.8 μm [25] in RNFL thickness have been reported. Our data have similar tendencies with such findings. However, these studies did not focus on the elderly population and the rate of decrement can also increase with aging. The age-related decrease in MCT may be explained by the effect of aging on ganglion cell complex (GCC) layer. Kim et al. [27] (N = 182, age range = 22 ~ 84 years) depicted a decrease of in GCC thickness (1.59 μm per decade), compared to other retinal layer thicknesses. According to Demirkaya et al. [26] (N = 121, mean age = 46.9 years), the thickness of pericentral GCL, which corresponds to the MCT area in our study, decreased significantly with increasing age. Since MCT encompasses the area with the thickest GCC layer, thinning of the GCC with aging may affect the MCT thickness directly, more than any other area. Once focused on only the elderly population, the rate of thinning may accelerate with aging.

In our study, subfoveal choroidal thickness decreased 2.77 μm yearly. It is known that physiologic functions of the choroid decrease with age, and histologic evaluations also show decreases in vascular density, overall luminal area, and diameter of the choriocapillary vessels [26–29]. Few studies exist which evaluate the overall thickness of the choroid [16]. Shin et al. (N = 40, mean age = 46.2 years) reported an annual reduction of choroidal thickness of the total ETDRS subfields of 0.97 μm [16]. The decrement rate in our study may have been overestimated, as we focused on the subfoveal region, which is regarded as the thickest part of the choroid. However, most studies measure the choroidal thickness on the subfoveal region [9, 30–34]. Employing such methods, choroidal thickness decreased 1.1 μm yearly, in autopsy eyes [31], while other in-vivo studies showed a reduction of 1.4–1.56 μm per year [33, 34]. However, these in-vivo studies were conducted in a relatively young-aged group of small sample size (n = 30~43), which may have diminished the reduction rate.

Our results showed that male gender was associated with a greater CFT and MCT, which corresponds to the area within the central 3 mm zone of the macula. This is consistent with findings by Adhi et al., which also depicted a thicker macular area in men in the same region using a SD-OCT system [19]. Many OCT studies describe gender differences in CFT or in other macular areas [18, 35–38]. However, few have examined the differences in the elderly population [18]. Studies that do divide young and old-aged groups define ‘old’ at a very early age (i.e. around the fifth decade) compared with our study which examines those 65 years of age or over. Such previous studies suggest no significant difference in the mean macular thickness in their so-called ‘old’ groups. Hormonal differences and cardiovascular risk factors have been suggested to explain differences of retinal thickness between genders, although the causal relationship remains unknown.

Genetic factors associated with choroidal thickness have been explored in patients with certain eye pathology. Hosoda et. al. reported a genome-wide association study that revealed *CFH* rs800292 to be related with choroidal thickness in central serous chorioretinopathy (CSC) patients [39]. Another study within the Japanese population showed that *CFH* rs1329428 also had association with choroidal thickness in PCV patients [10].

The key and novel finding of this study is the association of the *CFH* rs1061170 risk genotype with the thinning of subfoveal choroidal thickness in healthy elderly eyes. The subfoveal choroid was 14.7% thinner in the group containing the risk allele(C) of rs1061170 but free from other ocular diseases. This genetic population may be more susceptible to the development of choroid-related diseases, including AMD.

*CFH* rs1061170 is known as a major AMD-associated SNP, and is assumed to play a role in the pathogenesis of the disease [9], although details are as yet unclear. Choroidal thinning is also a characteristic finding of AMD and is considered to be an underlying pathology related to the etiology of AMD. Mullins et al. reported a 23.6% thinner choroid in eyes from donors homozygous for the *CFH* rs1061170 risk allele (CC) compared with non-risk allele homozygotes (TT) [40]. Histologic findings supported that changes in the choroidal vasculature, such as the loss of endothelial cells, occur early in AMD pathogenesis in eyes with high risk genotype [40].

From our findings, we speculate that in the aged population, *CFH* risk genotype induces choroidal thinning, which leads to the pathogenic process of AMD. We could further speculate on a putative vicious circle of *CFH* rs1061170, choroidal thinning and AMD. As CFH is an important negative regulator of the alternative complement pathway, its pro-inflammatory aspects have been discussed in age-associated inflammation [41]. *CFH* rs1061170 risk genotype carriers were thought to be predisposed to the chronic pro-inflammatory state which manifests as an increased risk of mortality in a population-based cohort of 90-year-olds in Finland [41]. In eyes, CFH is expressed in the RPE, drusen and choroidal capillaries. Thus, the lack or compromise of inflammatory modulation among *CFH* rs1061170 risk genotype carriers may affect the integrity of the choroid and result in pro-AMD features such as choroidal thinning.

*CFH* rs1061170 is known to be more highly associated with AMD in Caucasians (frequency of risk allele 35%), compared with Asians, including Koreans, due to the relatively low frequency (7.4%) of the risk allele in *CFH* rs1061170 in Asians [6]. However, we may speculate that if a risk-allele carrier, the incidence of AMD increases due to choroidal thinning and subsequent degeneration. Further studies on a larger population of patients and different ethnicities are needed to support such a hypothesis.

There are several limitations in our study: First, this is a prospective population-based study with cross-sectional analysis rather than a longitudinal analysis. To maximize our understanding regarding the aging retina and choroid, a longitudinal analysis must be performed, which we expect in further reports as this cohort study continues. Second, the low frequency of *CFH* rs1061170 risk allele inherent in the Asian population may have underestimated the effect of *CFH* rs1061170.

Despite such limitations, this population-based cohort study has been conducted in highly aged groups (over 65 years of age, mean age of 76.6 years) with a large number of participants, in which its strength lies. The study is also meaningful in that it suggests the significance of *CFH* rs1061170 risk genotype and its relationship with aging and chorio-retinal factors, not only in diseased eyes but also those of the healthy population. A vast number of randomly sampled participants all underwent imaging by a single SD-OCT device including EDI technique. This produces more reliable results with better quality compared to studies previously done with earlier-generation OCTs.

In conclusion, we have provided normative data for chorioretinal thickness in elderly population over 65 years of age using the SD-OCT system and the association with clinical, ophthalmic and genetic factors. In the healthy aging population, *CFH* rs1061170 is a significant genetic risk factor associated with choroidal thinning. Such findings may provide further insight into the pathogenesis of age-related macular degeneration as well as normal aging.
